# Attachment to Parents As a Moderator in the Association between Sibling Bullying and Depression or Suicidal Ideation among Children and Adolescents

**DOI:** 10.3389/fpsyt.2018.00072

**Published:** 2018-03-12

**Authors:** Jasmin Bar-Zomer, Anat Brunstein Klomek

**Affiliations:** ^1^Baruch Ivcher School of Psychology, Interdisciplinary Center (IDC), Herzliya, Israel

**Keywords:** bullying, sibling, attachment, depression, suicidal ideation, adolescence

## Abstract

Bullying is one of the most widespread phenomenon in childhood and adolescence. Interestingly, most research on bullying focuses on bullying at school and not on bullying among siblings at home. Sibling bullying is the most frequent form of repeated aggression that children experience in their lifetime. Furthermore, previous studies indicate that sibling bullying is associated with depression and self-harm behavior. However, the association between sibling bullying and suicidal ideation was never previously examined. Attachment to parents is one variable that can moderate the association between sibling bullying and depression/suicide ideation. To our knowledge, there is no existing study that examines the association between sibling bullying and attachment patterns. In addition, no previous study has examined the moderating role of attachment on the association between sibling bullying and depression or suicidal ideation among adolescents. The current study includes 279 Israeli students aged 10–17 (M = 13.5; SD = 1.98; 164, 58.8% females) who completed self-report questionnaires regarding school and sibling bullying, attachment to mother and father, depression, and suicidal ideation. The results indicated an association between bullying among siblings and school bullying. In addition, children and adolescents who were consistently involved in sibling bullying were at greater risk for depression and suicide ideation when compared to children and adolescents who were not involved in sibling bullying. A secure attachment to one’s father (but not to one’s mother) moderated the association between sibling bullying and depression/suicide ideation. It should be noted that when suicide ideation was examined above and beyond depression, attachment to one’s father did not moderate the association between sibling bullying involvement and suicide ideation. This finding indicates that depression plays a central role in the association between sibling bullying and suicide ideation. These results suggest that sibling bullying is a risk factor for depressive symptoms and suicide ideation and that secure attachment to one’s father may serve as a protective role. Future bullying prevention programs should include sibling bullying and encourage the increased availability of paternal emotional support. Other theoretical and applied implications for prevention of both sibling bullying and suicide are discussed.

## Introduction

Bullying is a subtype of aggression (1). Research on sibling relationships rarely defines sibling violence as a form of bullying (2). However, violence between siblings often meets the three criteria for bullying: a situation in which a child is exposed to unwanted aggression that is intended to induce fear, distress or harm; frequently repeats itself; and an unbalanced distribution of power between the aggressor and the aggressed (1, 3). Furthermore, an important literature review, conducted by Wolke et al. (4), suggests that we should relate to sibling bullying as we do to peer bullying. Therefore, it seems appropriate to categorize those involved in sibling bullying as either, victim, bully, or bully/victim (a child that is both, bully and victim), an acceptable categorization for those involved in peer or school bullying (5).

Despite the importance of sibling bullying, a dearth of research has examined this form of bullying (2, 4, 6). Wolke and Skew (7) reviewed research that examined the frequency of sibling bullying and found that about half of the children were involved in some form of sibling bullying (as bully, as victim, or as bully/victim) at a frequency of more than four times in 6 months. Of these children, 16–20% reported frequent sibling bullying involvement (i.e., once a week or more). Wolke et al.’s (4) review suggests that sibling bullying is the most frequent form of abuse. Children suffer from sibling bullying more than from abuse by parents, adult strangers, or peers (4, 7).

Research shows an association between sibling bullying and school bullying. Studies found that 50% of the children who reported sibling victimization also suffered from victimization at school (8, 9). Duncan (10) showed the reverse of this association such that children who suffered from school bullying reported higher levels of involvement with bullying at home, either as victim or as bully.

One of the negative ramifications of sibling bullying involvement is depression (11). In a longitudinal research study, Bowes et al. (11) found that children who were victims of sibling bullying at age 12 were at a double risk for depression, anxiety, and self-harm, compared to children who did not suffer from sibling bullying. In addition, results also showed that the higher the frequency of the sibling bullying, the higher the risk for psychological difficulties for the victim. Klomek et al. (12) found that adolescents who were victims of school bullying were at an increased risk for depression but also for suicidal ideation, and suicidal attempts. To the best of our knowledge, there are no previous studies, which have examined suicidal ideation among those involved in sibling bullying, compared to suicidal ideation among those who are not involved in sibling bullying.

Only a few studies have examined the association between familial characteristics and sibling bullying, while using the definition of sibling bullying (4). The existing research suggests an association between familial characteristics and involvement in sibling bullying. For example, children who had many conflicts with their parents, who were reared in more violent homes, who underwent parental abuse or whose mothers suffered from depression, were more likely to be involved in sibling bullying than children not involved in sibling bullying (7, 11, 13–17) but attachment was not examined. Attachment theory focuses on the long-term relationship dynamics between people, starting from the developing relationship between child and parent in the child’s first years of life (18, 19). The theory assumes this primary relationship to form the foundation for how the child perceives his relationship to the social world that surrounds him (20).

In recent decades, data from a range of research emphasized the association between attachment styles and the social and emotional development of children and adolescents. These associations, however, were not previously examined in relation to sibling bullying. There are a handful of studies, which examined the association between attachment style and sibling aggressiveness or violence. Results from these studies indicate that attachment styles toward one’s mother and father can dictate the relationships between siblings and influence an individual’s aggressive behavior (21–23). There is a high likelihood that siblings with insecure attachments toward their parents will respond with aggression, will engage in repeated conflicts, and will involve themselves in sibling bullying (6, 24, 25). Likewise, a small number of studies examined the association between attachment styles and involvement in school bullying (24, 26–29).

In summary, the associations between sibling bullying and suicidal ideation as well as the associations between sibling bullying and attachment to parents have not been previously examined. Moreover, the variables that moderate the association between sibling bullying and depression/suicidal ideation have yet to be examined. Therefore, the goal of the present research is to fill in these gaps. Our research hypotheses are as follows: (1) the likelihood for depression and suicidal ideation will be higher among children involved in sibling bullying compared to children not involved in sibling bullying; (2) children involved in sibling bullying will display less secure attachment styles toward their mothers and fathers compared to children not involved in sibling bullying; and (3) secure attachment styles toward one’s mother and father will moderate the association between involvement in sibling bullying and depression/suicidal ideation in the following way: among children with secure attachments with their mothers/fathers, there will be a weaker association between sibling bullying involvement and depression/suicidal ideation than among children with insecure attachments with their mothers/fathers.

## Materials and Methods

### Participants

Three hundred nineteen children and adolescents, ages 10–17, participated in the study. All participants were students in 5th through 12th grades in elementary schools, junior high schools, and high schools in Israel. Forty-nine participants were removed from the study due to incompletion of their questionnaires. Two hundred seventy-nine participants remained for the final analyses. These included 115 boys and 164 girls, in grades 5 to 12 (mean age—13.5, SD = 1.9).

### Research Measures

Five self-report and anonymous questionnaires were completed by participants.

#### Demographic Questionnaire

This questionnaire included questions regarding age, gender, grade level, and sibling composition (i.e., number, gender).

#### Sibling and Peer Bullying

The bullying questionnaire was used to measure the participants’ involvement in sibling and peer bullying. As in the original questionnaire (30), the first part of this questionnaire asked questions regarding school bullying and included 11 questions about bullying behaviors and 11 questions about victimization. Furthermore, this questionnaire is comprised of questions relating to type of bullying (e.g., physical bullying, “Hit, slapped or pushed you,” “Took your money”; verbal bullying, “Threatened to hit or harm you”), place of bullying (inside/outside of school, at home, at social media, *via* the cellphone), and the characteristics of the bully (individual/group, gender). In the second part of this questionnaire, we appropriated the questions for sibling bullying. The questions remained identical to how they were presented in the original questionnaire, just adapted for siblings. The frequency items were coded on a 4-point scale ranging from 1 to 4 (1 = *not at all*, 2 = *less than once a week*, 3 = *More than once a week*, 4 = *Most days*). An adolescent was considered involved in bullying if he/she marked frequent involvement (*more than once a week* or more) in any type of bullying or victimization involvement (physical, verbal, social, etc.). Earlier research shows good reliability (α = 0.79) for this questionnaire (31). In the present study, the reliability of this questionnaire was high, with an internal consistency score (alpha Cronbach) of α = 0.91 for the school bullying sub-scale, and an internal consistency score (alpha Cronbach) of α = 0.90 for the school victimization sub-scale. Likewise, the internal consistency score (alpha Cronbach) for the sibling bullying sub-scale was α = 0.90, and the internal consistency score (alpha Cronbach) for sibling victimization was α = 0.91.

#### Depression

In order to assess clinical depression, we used The *Mood and Feelings Questionnaire* [MFQ; (32)], a widely used questionnaire for identifying symptoms of depression among children and adolescents aged 8–18. The questionnaire includes 33 questions relating to the child’s/adolescent’s emotions, cognitions, and behaviors in the last 2 weeks. The questions require the child/adolescent to rank his/her answers on a scale of 0–2 (0 = *incorrect*, 1 = *sometimes correct*, 2 = *correct*). A sum score of 26 or above determines clinical depression, as suggested in research literature (33). Early findings show good validity and reliability (α = 0.87) for this questionnaire (34). The reliability of this questionnaire in the present study was high (α = 0.96).

#### Suicidal Ideation

Four items from the MFQ, all of which indicate suicidal ideation, were used to assess suicidal ideation. Three of the items reflect passive suicidal thinking (“I thought that there’s no reason to live,” “I thought about death,” and “I thought that my family is better off without me”), and one item reflects active suicidal thinking (“I thought to kill myself”). The differentiation between passive and active suicidal thinking is consistent with the Columbia Suicide Severity Rating Scale (35). Answers range on a scale from 0 to 2 (0 = *incorrect*, 1 = *sometimes correct*, 2 = *correct*). As accepted in research literature, each positive answer is indicative of suicidal ideation. The use of questions concerning suicidal ideation from a depression questionnaire has been done in earlier research (33). The reliability of these four questions in the present study was good (α = 0.85).

#### Attachment to Parents

Participants’ parent-child attachments were assessed with *The Attachment Security Style Scale* (36). This questionnaire measures one’s attachment style by using a Harter 4-point scale (“*There are children that* … *other children* …”). The child/adolescent is required to mark which of the statements is true regarding his/her relationship with his/her parent (e.g., “there are those that are not certain that they can trust their mother, but there are those for whom it is easy to trust their mothers”). Then, the child/adolescent is requested to decide if the statement is very true or pretty true with regard to himself/herself. The participants completed 2 editions of 15 items each, 1 regarding the mother and 1 regarding the father. Ratings for each participant were summed to form an attachment security score ranging from 15 to 60, with higher scores indicating a more secure relationship. Earlier findings showed high reliability and validity for this questionnaire (36, 37). The internal consistency (alpha Cronbach) score for the attachment to mother section was α = 0.84, while the internal consistency (alpha Cronbach) score for the attachment to father section was α = 0.87.

### Procedure

The pilot study included 91 children and adolescents who were recruited as a convenience sample. All parents signed an informed consent form. The children read an explanation of the study and, then, completed the questionnaires. The 228 additional participants were recruited *via* their schools. We first requested permission from the school principals. Once permission was granted, we sent printed and emailed letters to parents describing the study. Attached to the letter was a request for non-participation. 2 weeks later, during a class set aside for the study, the students who were approved for participation *via* their parents, completed the self-report questionnaires. The study was approved by the Israeli Ministry of Education and the ethical committee of the Interdisciplinary Center (IDC), Herzliya.

### Statistical Analyses

Chi-squared (χ^2^) tests were performed in order to examine dependency between sibling bullying involvement and school bullying involvement, depression, and/or suicidal ideation. Likewise, independent sample *T* tests were performed in order to examine if children who are involved in sibling bullying have less secure attachment styles (toward mother and father separately) compared to children who are not involved in sibling bullying. Eventually, a multiple logistic regression analysis was performed in order to examine if the level of secure attachment to one’s parent (mother and father separately) moderated the association between sibling bullying involvement and depression/suicidal ideation. In this analysis, the level of secure attachment (to mother and father separately), sibling bullying involvement, and the interaction between them were simultaneously entered as predictors. In accordance with Aiken et al. (38) and before conducting the regression analysis, we centered the values of the aforementioned variables (i.e., sibling bullying involvement and attachment). Considering the regression model for suicidal ideation yielded significance, we conducted a further analysis to examine if one’s attachment style moderates the association between involvement in sibling bullying and suicidal ideation while controlling for depression. All statistical analyses of the study data were performed on SPSS edition 21. Finally, considering that suicidal ideation was measured *via* four items from the MFQ for depression, we conducted a further analysis to be certain that the moderating model would remain significant, even after removing the four items from the depression measure and using them as a measure for suicidal ideation.

## Results

In the total sample, 86 participants were involved in sibling bullying (193 participants were not involved), while 92 participants were involved in school bullying (187 participants were not involved). Table 1 displays the frequency of involvement in school bullying and sibling bullying. In addition, 11.8% of the participants in the present sample met criteria for clinical depression and 19.7% of the participants presented either passive suicidal ideation or active suicidal ideation.

**Table 1:** The frequency of involvement in school bullying and sibling bullying.

	Percentage of the total sample which are involved in bullying			
	Victims	Bully	Bully victim	Any type of involvement
Sibling bullying	9.7% (*n* = 27)	4.3% (*n* = 12)	16.8% (*n* = 47)	30.8% (*n* = 86)
School bullying	13.2% (*n* = 37)	10.4% (*n* = 29)	9.3% (*n* = 26)	32.9% (*n* = 92)

### The Association between Involvement in Sibling Bullying and Involvement in School Bullying

A significant association was found between sibling and school bullying [χ^2^(1) = 25.73, *p* < 0.001]. Of the 86 participants involved in sibling bullying, 48 (55.8%) were involved in school bullying. By contrast, of the 193 participants not involved in sibling bullying, 46 (23.8%) were involved in school bullying. In other words, these results suggest that there is a significant association between involvement in school bullying and involvement in sibling bullying. Children involved in sibling bullying were 2.3 times more likely to suffer from school bullying than children not involved in sibling bullying.

### The Association between Involvement in Sibling Bullying and Depression and Suicidal Ideation

A significant association between sibling bullying involvement and depression was found [χ^2^(1) = 15.34, *p* < 0.001]. Of the 86 participants involved in sibling bullying, 20 (23.2%) met criteria for clinical depression. By contrast, of the 193 participants uninvolved in sibling bullying, only 12 (6.2%) met criteria for clinical depression. In other words, these results suggest that there is a significant association between involvement in sibling bullying and clinical depression. Children involved in sibling bullying were 3.7 times more likely to suffer from clinical depression than children not involved in sibling bullying.

In addition, a significant association between sibling bullying involvement and suicidal ideation was found [χ^2^(1) = 10.436, *p* < 0.005]. Of the 86 participants involved in sibling bullying, 26 (30.2%) experience suicidal ideation. In contrast, of the 193 participants uninvolved in sibling bullying, only 25 (12.9%) experienced suicidal ideation. In other words, these results suggest that there is a significant association between involvement in sibling bullying and suicidal ideation such that children involved in sibling bullying were 2.3 times more likely to suffer from suicidal ideation than children not involved in sibling bullying.

### The Association between Sibling Bullying Involvement and Attachment Style

We found a significant difference in levels of secure attachment to mothers and fathers between those involved in sibling bullying and those not involved in sibling bullying [*Mother*: *t*(232) = 4.09, *p* < 0.001; *Father*: *t*(224) = 3.32, *p* < 0.005]. Participants involved in sibling bullying have less secure attachments to their mothers (M = 43.41) than do participants uninvolved in sibling bullying (M = 48.19). Likewise, participants involved in sibling bullying have less secure attachments to their fathers (M = 41.69) than do participants uninvolved in sibling bullying (M = 46.36).

### Attachment to Mother As a Moderating Variable on the Association between Sibling Bullying Involvement and Depression/Suicidal Ideation

The regression model that examined attachment to mother as moderating the association between sibling bullying and depression, while controlling for gender, was found to be significant (−2LL = 119.53, *p* < 0.001, Nagelkerke *R*^2^ = 0.24, Cox & Snell *R*^2^ = 0.11). The analysis resulted in a positive association between gender and depression such, that girls had a 4.76 times higher chance for depression than did boys (*B* = 1.56, *Z* = 2.37, OR = 4.76, *p* < 0.05). Attachment style to mother was *almost* significantly associated with depression (*B* = −0.08, *Z* = 1.96, OR = 0.92, *p* = 0.0504). In addition, involvement in sibling bullying was found to be positively and significantly associated with depression (*B* = 1.38, *Z* = 2.52, OR = 3.97, *p* < 0.05) such, that participants involved in sibling bullying have a 3.97 times higher chance for depression than do participants uninvolved in sibling bullying. Finally, an interaction effect between level of secure attachment to mother and bullying involvement was found to be insignificant (*B* = 0.02, OR = 3.73, *Z* = 0.29, ns). After conducting the above analyses, we performed an additional multiple logistic regression analysis in order to examine if gender influences the way in which attachment style to mother moderates the association between sibling bullying involvement and depression. This model did not appear significant, a likely result of the sample size.

A multiple logistic regression analysis was also performed to examine if the level of secure attachment to the mother moderates the association between sibling bullying involvement and suicidal ideation. This model was found to be significant (−2LL = 201.12, *p* < 0.005, Nagelkerke *R*^2^ = 0.12, Cox & Snell *R*^2^ = 0.07). This analysis resulted in a positive association between gender and suicidal ideation such, that girls have a 2.66 times higher chance for suicidal ideation than do boys (*B* = 0.98, *Z* = 2.36, OR = 2.66, *p* < 0.05). Likewise, analysis results showed that a secure attachment style to one’s mother is negatively and significantly associated with suicidal ideation (*B* = −0.05, *Z* = −2.15, OR = 0.96, *p* < 0.05). In other words, the more secure one’s attachment is to his/her mother, the lower the chance for suicidal ideation. Involvement in sibling bullying was not found to be significantly associated with suicidal ideation (*B* = 0.68, *Z* = 1.80, OR = 1.98, ns). Finally, an interaction effect between attachment to mother and involvement in sibling bullying was found to be insignificant (*B* = −0.01, *Z* = −0.13, OR = 1.88, ns). In other words, attachment to one’s mother did not moderate the association between sibling bullying involvement and suicidal ideation. After the above analyses, we performed an additional multiple logistic regression analysis in order to examine if gender influences the way in which attachment style to one’s mother moderates the association between sibling bullying involvement and suicidal ideation. This model did not appear significant, a likely result of the sample size.

### Attachment to Father As a Moderating Variable on the Association between Sibling Bullying Involvement and Depression/Suicidal Ideation

**Figure 1:**
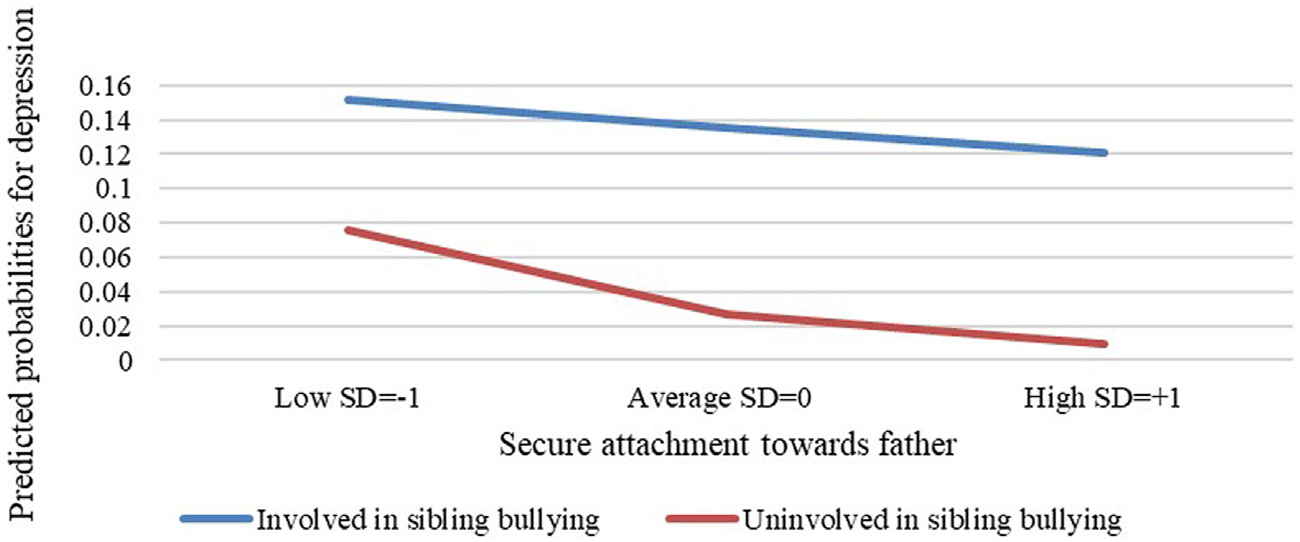
Attachment to one’s father as a moderating variable in the association between involvement in sibling bullying and depression.

The multiple logistic regression model performed to examine if the level of secure attachment to one’s father moderates the association between involvement in sibling bullying and depression was found to be significant (−2LL = 114.49, *p* < 0.001, Nagelkerke *R*^2^ = 0.26, Cox & Snell *R*^2^ = 0.11). This analysis resulted in a positive association between gender and depression such, that girls have a 5.5 times higher chance for depression than do boys (*B* = 1.71, *Z* = 2.42, OR = 5.5, *p* < 0.05). Attachment style to one’s father was found to be negatively and significantly associated with depression (*B* = −0.11, *Z* = −3.11, OR = 0.9, *p* < 0.005). Meaning, the more secure one’s attachment is to his/her father, the lower the chance for depression. In addition, involvement in sibling bullying was found to be positively and significantly associated with depression (*B* = 1.73, *Z* = 2.96, OR = 5.67, *p* < 0.005) such that participants involved in sibling bullying have a 5.67 times higher chance for depression than participants uninvolved in sibling bullying. Finally, an interaction effect between level of secure attachment to one’s father and involvement in sibling bullying was found to be significant (*B* = 0.09, Wald = 4.37, OR = 5.60, *p* < 0.05). In other words, attachment to one’s father moderates the association between sibling bullying involvement and depression such, that the more secure one’s attachment is to his/her father, the weaker the association between sibling bullying involvement and depression (Figure 1).

After the above analyses, we performed another multiple logistic regression analysis in order to examine if gender influences the way in which attachment style to one’s father moderates the association between involvement in sibling bullying and depression. This model did not appear significant, a likely result of the sample size.

In addition, in order to be certain that the moderating model for depression would remain significant after separating the variables for depression and suicidal ideation; we examined the depression measure after removing the four items used for the suicidal ideation measure. We kept the threshold value for the depression questionnaire at 26, as a stringent criterion for determining clinical depression. A multiple logistic regression analysis was performed in order to examine if the level of secure attachment to one’s father moderates the association between involvement in sibling bullying and depression (without the four items used to measure suicidal ideation). This moderating model appeared significant, meaning that attachment to one’s father moderates the association between sibling bullying involvement and depression such, that the more secure one’s attachment is to his/her father, the weaker the association between involvement with sibling bullying and depression (*B* = 0.11, Wald = 6.73, OR = 2.69, *p* < 0.05).

**Figure 2:**
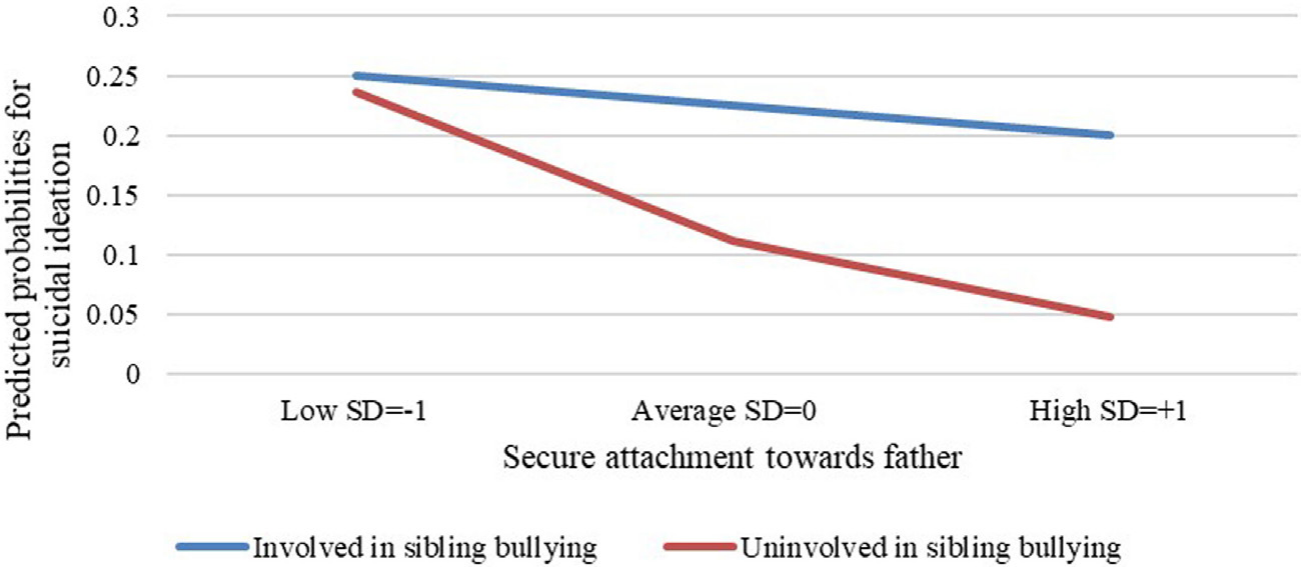
Attachment to one’s father as a moderating variable in the association between involvement in sibling bullying and suicidal ideation.

The multiple logistic regression analysis, which examined if the level of secure attachment with one’s father moderates the association between sibling bullying involvement and suicidal ideation, was found to be significant (−2LL = 186.94, *p* < 0.001, Nagelkerke *R*^2^ = 0.18, Cox & Snell *R*^2^ = 0.11). This analysis resulted in a significant and positive association between gender and suicidal ideation such that girls have a three times higher chance for suicidal ideation than do boys (*B* = 1.11, *Z* = 2.53, OR = 3.02, *p* < 0.05). Likewise, it was found that a secure attachment style to one’s father is negatively and significantly associated with suicidal ideation (*B* = −0.06, *Z* = −3.48, OR = 0.94, *p* < 0.001). In other words, the more secure one’s attachment is to his/her father, the lower the chance for suicidal ideation. Sibling bullying involvement was found to be significantly related to suicidal ideation (*B* = 0.84, *Z* = 2.14, OR = 2.31, *p* < 0.05), meaning that participants involved in sibling bullying have a 2.3 times higher chance for suicidal ideation than do participants uninvolved in sibling bullying. Finally, an interaction effect between level of secure attachment to one’s father and involvement in sibling bullying was found to be significant (*B* = 0.07, Wald = 0.04, OR = 2.35, *p* < 0.05). In other words, attachment to one’s father moderates the association between involvement in sibling bullying and suicidal ideation such, that the more secure the attachment is to one’s father, the weaker the association between involvement in sibling bullying and suicidal ideation (Figure 2). After this analysis, we performed an additional multiple logistic regression analysis in order to examine if gender influences the way in which attachment to one’s father moderates the association between involvement in sibling bullying and suicidal ideation. This model did not appear significant, again, a likely result of the sample size.

In order to examine if one’s attachment style to his/her father moderates the association between involvement in sibling bullying and suicidal ideation beyond depression, we conducted a further analysis. In this analysis, we entered depression as a control variable and, as expected, an interaction effect between level of secure attachment to one’s father and involvement in sibling bullying was found to be not significant (*B* = 0.05, Wald = 1.46, OR = 1.06, *p* = 0.16). Furthermore, sibling bullying involvement was not significantly associated with suicidal ideation (*B* = 0.84, *Z* = 1.79, OR = 2.12, *p* = 0.07). In other words, depression seems to play a central role in suicidal ideation and explains a large amount of its variance.

## Discussion

The findings of the present study suggest an association between involvement in sibling bullying and involvement in school bullying. The findings also suggest that involvement in sibling bullying is associated with depression and suicidal ideation. In addition, attachment to one’s father (and not to one’s mother) moderates the association between involvement in sibling bullying and depression/suicidal ideation among children/adolescents aged 8–18. However, the association between sibling bullying and suicidal ideation is not significant when depression is controlled for. In the same way, attachment to one’s father does not appear to moderate the association between sibling bullying involvement and suicidal ideation when controlling for depression.

The findings, which support an association between sibling bullying involvement and school bullying involvement, are consistent with previous findings (7, 8). Earlier research assumes that behavioral structures learned at home and between siblings are transferred to relationships with peers (16). For example, studies have shown that children who are victims of sibling bullying are more likely to be victimized at school [e.g., Ref. (8)]. Victims of sibling bullying learn from their sibling interactions that they have little value and that they are powerless in the face of violence from others. As a result, they are likely to develop a submissive, non-assertive, and avoidant interpersonal style, which increases their chance for involvement in school bullying (10, 15). Likewise, school bullying can influence bullying at home. For example, research shows that school bullies display more negative emotions toward their brothers than do children who are uninvolved in bullying (39).

Our findings that individuals involved in sibling bullying are 3.6 times more likely for depression than are individuals not involved in bullying are consistent with previous studies (4, 11). One possible explanation for this association is that bullying between siblings is often a continuous situation, with those involved trapped in their own home environments without any possibility of avoiding or escaping the situation (11). A child is exposed to violence under his own roof while the parental figures which are responsible to protect, defend, support, and create guidelines are missing or not satisfactorily involved. A child involved in bullying is likely to develop a sense of helplessness and lack of security, which are likely to increase depression. Sibling bullying can therefore become a very harmful experience and present as a risk factor for emotional and psychological issues (11). The finding that participants involved in sibling bullying are at a 2.3 times higher risk for suicidal ideation than are participants uninvolved in sibling bullying, is the first of its kind in the field of sibling bullying. This finding is consistent with earlier studies which found an association between school bullying involvement and suicidal ideation (12, 40), independent of depression (41). The results which indicated an insignificant association between sibling bullying involvement and suicidal ideation once depression was entered into the model suggest that depression explains a large portion of this association.

The finding that participants involved in sibling bullying have less secure attachments to their mothers and fathers than do participants not involved in sibling bullying is in line with earlier findings regarding the association between involvement in school bullying and attachment styles (24, 29). The child’s relationships with his/her parents are the first relationships the child will experience and through them he/she will learn what to expect from other relationships, how to behave in other relationships, and will develop useful (or not) interpersonal skills (18, 42). Children who suffer from difficulties in their attachment with their parents tend to not rely on others, display lower levels of empathy and concern for others, present low self-esteem and a need for affirmations from others, and show a wide range of adaptation difficulties across the life-span (6). Children and adolescents with these characteristics are at a high risk for many conflicts and bullying, including sibling bullying (6, 24, 25).

The finding that participants with a more secure attachment style with their fathers (not with their mothers) have a weaker association between involvement in sibling bullying and depression/suicidal ideation than participants with a less secure attachment style with their fathers, is consistent with the results Nikiforou et al.’s (28) study which found that attachment to one’s mother was less contributing to victimization involvement than attachment to one’s father. However, these results differ from the results of Klomek et al.’s (43) study, which suggest a significant association between attachment style to one’s mother and the likelihood for victimization involvement at school. The difference between Klomek et al.’s (43) finding and the present study’s finding may be explained by the fact that Klomek et al. (43) examined only peer victimization, while the present study examined all types of involvement in sibling bullying. It seems that attachment style to one’s mother may have a different influence on the different roles involved in bullying (bully, victim, bully/victim). In addition, there seems to be a difference between involvement in bullying at home and in school.

Our findings are also consistent with the results of Desjardins and Leadbeater (44) which found that emotional support from the father (but not from mother) moderated the association between victims and depressive symptoms over time. It is possible that each parent provides different emotional support styles. Future research is needed to assess differences in the strategies that parents use to support their adolescents. Another potential explanation for why one’s attachment style to his/her mother does not moderate the association between involvement in sibling bullying and depression/suicidal ideation may be that the adolescent feels the need to protect his/her mother’s feelings (45). It may be that even if an individual holds a secure attachment with his/her mother, avoiding involving her out of fear for her emotional reaction narrows her ability to influence and moderate the association between involvement in sibling bullying and depression/suicidal ideation.

The present study has a few limitations. First, a majority (71.5%) of the research data was collected from one Israeli school and the sample size was relatively small. This limits our ability to generalize the findings. Second, the study is correlational, thus causality cannot be deduced. Third, all research variables were solely based on self-report questionnaires. Moreover, our measure for suicidal ideation was based on four items from the MFQ questionnaire for depression. Assessing suicidal ideation in this way limits our ability to assess and identify suicidal risk because we need to distinguish between suicidal ideation and suicidal behavior (35). Future studies should examine suicidal behavior and expand the measurement for suicidal ideation *via* a distinct tool. Finally, possible differences between different types of sibling bullying involvement (victim, bully, bully/victim) were not examined. Future research should examine if involvement in sibling bullying as a bully/victim is significantly different from involvement in sibling bullying as a bully or as a victim. Furthermore, the definition of the bully/victim type in the field of sibling bullying is still complicated, as it might include a sibling which both bullies and victimized by the same sibling(s). This raises questions regarding the criteria necessary for defining bullying, an imbalance of power. Future research should continue exploring the sibling bully/victim type.

Despite these limitations, the present study provides important clinical implications. The present study suggests that the assessment of sibling bullying involvement must be included as a risk factor in prevention protocols and in questionnaires, which identify depression and suicidality among adolescents. Likewise, parents, educators and children must be able to distinguish between fighting, violence, and sibling bullying, since involvement in sibling bullying is associated with psychopathology and perhaps, even, suicide ideation. In addition, it is critical for parents and, specifically, for fathers, to understand their role in their child’s relationships with his/her siblings as well as the emotional aspects of such (44, 46). Future interventions and programs for the prevention of involvement in sibling bullying should enhance the father’s emotional support capacity. It is important that future studies continue to examine the mother’s role in the context of sibling bullying and depression/suicide ideation.

## Author Contributions

JB-Z conceptualized the topic as part of her MA studies in clinical psychology. JB-Z collected the data, analyzed the data, and drafted the first draft of the manuscript. AB-K supervised all stages of the work, including conceptualization, recruitment, analysis, and writing. JB-Z and AB-K are jointly accountable for the content of the work, ensuring that all aspects related to accuracy or integrity of the study are investigated and resolved in an appropriate way.

## Conflict of Interest Statement

The authors declare that the research was conducted in the absence of any commercial or financial relationships that could be construed as a potential conflict of interest. The reviewer LA and handling editor declared their shared affiliation.

## References

[1] Olweus D Bully/victim problems among schoolchildren: basic facts and effects of a school based intervention program. In: Pepler D Rubin K, editors. The Development and Treatment of Childhood Aggression. Hillsdale, NJ: Erlbaum (1991). p. 411–48.

[2] Hoetger LA Hazen KP Brank EM All in the family: a retrospective study comparing sibling bullying and peer bullying. J Fam Violence (2015) 30(1):103–11. 10.1007/s10896-014-9651-0

[3] Olweus D Bullying at School: What We Know and What We Can Do. Oxford, UK: Blackwell Publishers (1993).

[4] Wolke D Tippett N Dantchev S. Bullying in the family: sibling bullying. Lancet Psychiatry (2015) 2(10):917–29. 10.1016/S2215-0366(15)00262-X 26462226

[5] Haynie DL Nansel T Eitel P Crump AD Saylor K Yu K Bullies, victims, and bully/victims: distinct groups of at-risk youth. J Early Adolesc (2001) 21(1):29–49. 10.1177/0272431601021001002

[6] Kim J Kim E. Bullied by siblings and peers the role of rejecting/neglecting parenting and friendship quality among Korean children. J Interpers Violence (2016):1–24. 10.1177/0886260516659659 27436089

[7] Wolke D Skew AJ. Bullying among siblings. Int J Adolesc Med Health (2012) 24(1):17–25. 10.1515/ijamh.2012.004 22909908

[8] Wolke D Samara MM Bullied by siblings: association with peer victimization and behavior problems in Israeli lower secondary school children. J Child Psychol Psychiatry (2004) 45(5):1015–29. 10.1111/j.1469-7610.2004.t01-1-00293.x 15225343

[9] Sapouna M Wolke D Vannini N Watson S Woods S Schneider W Individual and social network predictors of the short-term stability of bullying victimization in the United Kingdom and Germany. Br J Educ Psychol (2012) 82(2):225–40. 10.1111/j.2044-8279.2011.02022.x 22583088

[10] Duncan RD Peer and sibling aggression: an investigation of intra- and extra-familial bullying. J Interpers Violence (1999) 14(8):871–86. 10.1177/088626099014008005

[11] Bowes L Wolke D Joinson C Lereya ST Lewis G. Sibling bullying and risk of depression, anxiety, and self-harm: a prospective cohort study. Pediatrics (2014) 134(4):e1032–9. 10.1542/peds.2014-0832 25201801

[12] Klomek AB Sourander A Niemelä S Kumpulainen K Piha J Tamminen T Childhood bullying behaviors as a risk for severe suicide attempts and completed suicides. J Am Acad Child Adolesc Psychiatry (2009) 48(3):254–61. 10.1097/CHI.0b013e318196b91f 19169159

[13] Button DM Gealt R High risk behaviors among victims of sibling violence. J Fam Violence (2010) 25(2):131–40. 10.1007/s10896-009-9276-x

[14] Eriksen S Jensen V. A push or a punch: distinguishing the severity of sibling violence. J Interpers Violence (2009) 24(1):183–208. 10.1177/0886260508316298 18417730

[15] Menesini E Camodeca M Nocentini A. Bullying among siblings: the role of personality and relational variables. Br J Dev Psychol (2010) 28(4):921–39. 10.1348/026151009X479402 21121475

[16] Tippett N Wolke D. Aggression between siblings: associations with the home environment and peer bullying. Aggress Behav (2015) 41(1):14–24. 10.1002/ab.21557 25187483

[17] Updegraff KA Thayer SM Whiteman SD Denning DJ McHale SM Relational aggression in adolescents’ sibling relationships: links to sibling and parent-adolescent relationship quality. Fam Relat (2005) 54(3):373–85. 10.1111/j.1741-3729.2005.00324.x

[18] Bowlby J Attachment, Vol. 1 of Attachment and Loss. New York: Basic Books (1969).

[19] Bowlby J Attachment and loss: retrospect and prospect. Am J Orthopsychiatry (1982) 52(4):664–78. 10.1111/j.1939-0025.1982.tb01456.x 7148988

[20] Sroufe LA Coffino B Carlson EA. Conceptualizing the role of early experience: lessons from the Minnesota longitudinal study. Dev Rev (2010) 30(1):36–51. 10.1016/j.dr.2009.12.002 20419077 PMC2857405

[21] Grossmann K Grossmann KE Kindler H Zimmermann P A wider view of attachment and exploration: the influence of mothers and fathers on the development of psychological security from infancy to young adulthood. 2nd ed In: Cassidy J Shaver PR, editors. Handbook of Attachment: Theory, Research, and Clinical Applications. New York: Guilford Press (2008). p. 857–79.

[22] Houston J Grych J Maternal attachment buffers the association between exposure to violence and youth attitudes about aggression. Journal of Clinical Child & Adolescent Psychology (2015) 45(5):605–13. 10.1080/15374416.2014.987380 25658051

[23] Volling BL Belsky J The contribution of mother–child and father–child relationships to the quality of sibling interaction: a longitudinal study. Child Dev (1992) 63(5):1209–22. 10.2307/1131528 1446550

[24] Eliot M Cornell DG Bullying in middle school as a function of insecure attachment and aggressive attitudes. Sch Psychol Int (2009) 30(2):201–14. 10.1177/0143034309104148

[25] Whiteman SD McHale SM Soli A Theoretical perspectives on sibling relationships. J Fam Theory Rev (2012) 3(2):124–39. 10.1111/j.1756-2589.2011.00087.x PMC3127252 21731581

[26] Coleman PK Perceptions of parent–child attachment, social self-efficacy, and peer relationships in middle childhood. Infant Child Dev (2003) 12(4):351–68. 10.1002/icd.316

[27] Kõiv K Attachment styles among bullies, victims and uninvolved adolescents. Psychol Res (2012) 2(3):160–5. 10.17265/2159-5542/2012.03.003

[28] Nikiforou M Georgiou SN Stavrinides P Attachment to parents and peers as a parameter of bullying and victimization. J Criminol (2013) 2013:e484871 10.1155/2013/484871

[29] Walden LM Beran TN Attachment quality and bullying behavior in school-aged youth. Can J Sch Psychol (2010) 25(1):5–18. 10.1177/0829573509357046

[30] Sourander A Brunstein Klomek A Ikonen M Lindroos J Luntamo T Koskelainen M Psychosocial risk factors associated with cyberbullying among adolescents: a population-based study. Arch Gen Psychiatry (2010) 67(7):720–8. 10.1001/archgenpsychiatry.2010.79 20603453

[31] Klomek AB Kleinman M Altschuler E Marrocco F Amakawa L Gould MS Suicidal adolescents’ experiences with bullying perpetration and victimization during high school as risk factors for later depression and suicidality. J Adolesc Health (2013) 53(1):S37–42. 10.1016/j.jadohealth.2012.12.008 23790199

[32] Angold A Costello EJ Messer SC Pickles A Development of a short questionnaire for use in epidemiological studies of depression in children and adolescents. Int J Methods Psychiatr Res (1995) 5(4):237–49.

[33] Costello EJ Angold A Scales to assess child and adolescent depression: checklists, screens, and nets. J Am Acad Child Adolesc Psychiatry (1988) 27(6):726–37. 10.1097/00004583-198811000-00011 3058677

[34] Sund AM Larsson B Wichstrøm L Depressive symptoms among young Norwegian adolescents as measured by the Mood and Feelings Questionnaire (MFQ). Eur Child Adolesc Psychiatry (2001) 10(4):222–9. 10.1007/s007870170011 11794547

[35] Posner K Brown GK Stanley B Brent DA Yershova KV Oquendo MA The Columbia–Suicide severity rating scale: initial validity and internal consistency findings from three multisite studies with adolescents and adults. Am J Psychiatry (2011) 168(12):1266–77. 10.1176/appi.ajp.2011.10111704 22193671 PMC3893686

[36] Kerns KA Klepac L Cole AK Peer relationships and preadolescents’ perceptions of security in the mother-child relationship. Dev Psychol (1996) 32(3):457–66. 10.1037/0012-1649.32.3.457

[37] Granot D Maysless O Attachment security and adjustment to school in middle childhood. Int J Behav Dev (2001) 25(6):530–41. 10.1080/01650250042000366

[38] Aiken LS West SG Reno RR Multiple Regression: Testing and Interpreting Interactions. Thousand Oaks, CA: Sage Publications (1991).

[39] Connolly I O’Moore M Personality and family relations of children who bully. Pers Individ Dif (2003) 35(3):559–67. 10.1016/S0191-8869(02)00218-0

[40] Bannink R Broeren S van de Looij–Jansen PM de Waart FG Raat H. Cyber and traditional bullying victimization as a risk factor for mental health problems and suicidal ideation in adolescents. PLoS One (2014) 9(4):e94026. 10.1371/journal.pone.0094026 24718563 PMC3981739

[41] Kaltiala-Heino R Rimpelä M Marttunen M Rimpelä A Rantanen P. Bullying, depression, and suicidal ideation in Finnish adolescents: school survey. BMJ (1999) 319(7206):348–51. 10.1136/bmj.319.7206.348 10435954 PMC28187

[42] Duncan RD The impact of family relationships on school bullies and their victims. In: Espelage DL Swearer SM, editors. Bullying in American Schools. Mahwah, NJ: Erlbaum (2004). p. 227–44.

[43] Klomek AB Kopelman-Rubin D Al-Yagon M Berkowitz R Apter A Mikulincer M Victimization by bullying and attachment to parents and teachers among student who report learning disorders and/or attention deficit hyperactivity disorder. Learn Disabil Q (2016) 39(3):182–90. 10.1177/0731948715616377

[44] Desjardins TL Leadbeater BJ. Relational victimization and depressive symptoms in adolescence: moderating effects of mother, father, and peer emotional support. J Youth Adolesc (2011) 40(5):531–44. 10.1007/s10964-010-9562-1 20577897 PMC4905762

[45] Williams SK Kelly FD Relationships among involvement, attachment, and behavioral problems in adolescence: examining fathers’ influence. J Early Adolesc (2005) 25(2):168–96. 10.1177/0272431604274178

[46] Sarkadi A Kristiansson R Oberklaid F Bremberg S. Fathers’ involvement and children’s developmental outcomes: a systematic review of longitudinal studies. Acta Paediatr (2008) 97(2):153–8. 10.1111/j.1651-2227.2007.00572.x 18052995

